# Detection of coronavirus-2 by real-time reverse transcription polymerase chain reaction in conjunctival swabs from patients with severe form of Coronavirus disease 2019 in São Paulo, Brazil

**DOI:** 10.6061/clinics/2021/e2913

**Published:** 2021-07-08

**Authors:** Mariana Akemi Matsura Misawa, Tatiana Tanaka, Tomás Minelli, Pedro Gomes Oliveira Braga, Juliana Mika Kato, Michele Soares Gomes Gouvêa, João Renato Rebello Pinho, Joyce Hisae Yamamoto

**Affiliations:** I Departamento de Oftalmologia (LIM 33), Hospital das Clinicas HCFMUSP, Faculdade de Medicina, Universidade de Sao Paulo, Sao Paulo, SP, BR.; II Instituto de Ensino e Pesquisa Santa Cruz (IPESC), Sao Paulo, SP, BR.; III Laboratorio de Gastroenterologia e Hepatologia Tropical (LIM 07), Departamento de Gastroenterologia, Hospital das Clinicas HCFMUSP, Faculdade de Medicina, Universidade de Sao Paulo, Sao Paulo, SP, BR.

**Keywords:** COVID-19, Conjunctiva, SARS-CoV-2

## Abstract

**OBJECTIVES:**

To test conjunctival swabs from patients with laboratory-confirmed severe forms of coronavirus disease 2019 (COVID-19) for the presence of SARS-CoV-2 on real-time reverse-transcription polymerase chain reaction (rRT-PCR).

**METHODS:**

Fifty conjunctival swabs were collected from 50 in-patients with laboratory-confirmed severe forms of COVID-19 at the largest teaching hospital and referral center in Brazil (HCFMUSP, São Paulo, SP). The samples were tested for SARS-CoV-2 on rRT-PCR with the primers and probes described in the CDC protocol which amplify the region of the nucleocapsid N gene (2019_nCoV_N1 and 2019_nCoV_N2) of SARS-CoV-2 RNA and compared with naso/oropharyngeal swabs collected within 24 hours of the conjunctival swabs.

**RESULTS:**

Five conjunctival samples (10%) tested positive (amplification of the N1 and N2 primer/probe sets) while two conjunctival samples (4%) yielded inconclusive results (amplification of the N1 primer/probe set only). The naso/oropharyngeal swabs were positive for SARS-CoV-2 on rRT-PCR in 34 patients (68%), negative in 14 (28%) and inconclusive in 2 (4%). The 5 patients with positive conjunctival swabs had positive (n=2), negative (n=2) or inconclusive (n=1) naso/oropharyngeal swabs on rRT-PCR. Patients with negative or inconclusive naso/oropharyngeal swabs had the diagnosis of COVID-19 confirmed by previous positive rRT-PCR results or by serology.

**CONCLUSION:**

This is the first study to present conjunctival swab rRT-PCR results for SARS-CoV-2 in a Brazilian population. In our sample of 50 patients with severe forms of COVID-19, 10% had positive conjunctival swabs, most of which were correlated with positive naso/oropharyngeal rRT-PCR results.

## INTRODUCTION

The ongoing pandemic of the novel coronavirus disease 2019 (COVID-19) is caused by the highly contagious severe acute respiratory syndrome coronavirus 2 (SARS-CoV-2) which primarily targets lung tissue and may cause pneumonia, respiratory distress, multiple organ failure and death (1). Despite typically presenting as a respiratory illness, extrapulmonary manifestations have been reported, some of which affecting the central nervous system, the gastrointestinal tract and the eye. Ocular manifestations of COVID-19 are reported to occur in 0.8-31.6% of patients with laboratory-confirmed COVID-19 and may present prior to the onset of pneumonia (1-3). In a large Chinese cohort of 1,099 infected patients, 9 (0.8%) had conjunctival congestion, and 4 (2.3%) of the patients with severe illness had conjunctivitis (1). The presence of conjunctivitis has been associated with severe forms of the disease (4,5).

The detection of SARS-CoV-2 in ocular samples on real time reverse-transcription polymerase chain reaction (rRT-PCR) has already been reported, even in cases with naso/oropharyngeal swabs testing negative on rRT-PCR (6,7). The positivity rate varies from 0 to 28.6% (8-10). More attention has been given to the possibility of ocular infection after healthcare workers were shown to have been infected despite being fully gowned with protective suits and N95 respirator (11,12). The virus has also been detected in the conjunctiva of asymptomatic patients (6,7). Patients with ocular symptoms were at first believed to be more likely to have positive conjunctival swabs than patients with no eye discharge (13), but some authors have reported similar rates of positivity in patients with and without symptoms, showing that viral shedding is not necessarily correlated with inflammation (14,15). On the other hand, higher rates of positivity have been associated with more severe forms of COVID-19 (7,14,16,17).

Brazil had the first case of COVID-19 positivity reported on February 25 2020, and the first death associated with COVID-19 occurred on March 17 2020. At the time of writing (February 3, 2021), 227,563 deaths have been ascribed to the virus. São Paulo is the most affected state with 1,807,009 confirmed cases and 53,704 deaths during the same period (18). We believe a more in-depth investigation of tear and conjunctival secretions as a potential source of infection would contribute significantly to diagnosis and control. PCR is the most widely used COVID-19 detection method among ophthalmologists, and conjunctival swabs have become the gold standard of sampling (17). The purpose of this study was to use rRT-PCR to evaluate conjunctival swabs collected from a large series of patients with severe forms of COVID-19. To our knowledge, this is the first Brazilian study to systematically analyze ocular samples for the presence of SARS-CoV-2.

### Patients and methods

A cross-sectional study was conducted between August 10 and December 18 2020 at the largest teaching hospital and referral center in Brazil (HCFMUSP, São Paulo), with more than 300 adult ICU beds dedicated to COVID-19 patients during the peak of the pandemic. The study protocol was approved by the Institutional Ethics Committee (CAAE 34937020.4.3001.8098) and informed consent was obtained prior to the study procedures. The sample consisted of adult patients with severe, critical and laboratory-confirmed COVID-19 infection. Severe disease was defined as clinical signs of pneumonia plus one or more of the following findings: i) resting oxygen saturation (SpO_2_) of less than 94% (range: 90-94) on room-air pulse oximetry, ii) arterial partial pressure of oxygen (PaO_2_) to fractional inspired oxygen (FiO_2_) ratio of less than 300 mmHg, iii) respiratory rate of 30 or more breaths per minute, and iv) pulmonary infiltrate occupying more than 50% of the lung parenchyma. Critical disease was defined as respiratory failure, shock and/or multiple organ failure. All the patients in the sample were hospitalized for 1.7±0.64 days (range: 1-4) before the conjunctival sample was collected. Tears and conjunctival samples were collected from the lower conjunctival fornix by sweeping the eye discharge 2 or 3 times using sterile flocked nylon swabs without topical anesthesia. The tip of the swab was placed in a sterile tube containing 200-250 µL saline solution. After being collected randomly from one eye of each patient within 24 hours of the collection of naso/oropharyngeal samples by qualified healthcare professionals wearing full personal protective equipment, the ocular samples were immediately refrigerated, sent to the laboratory and stored at -80°C until processing. The diagnosis of SARS-CoV-2 was confirmed for all patients with naso/oropharyngeal swabs testing positive on rRT-PCR, or by serology.

### Real-time reverse-transcriptase polymerase chain reaction analysis

Samples were tested for SARS-CoV-2 using rRT-PCR. RNA was extracted with the QIAamp Viral RNA Mini Kit (Qiagen, Hilden, Germany). Briefly, 1,120 μL lysis buffer AVL with RNA carrier was added to the sample tube containing saline solution (200-250 µL). RNA was purified according to the manufacturer’s protocol using a high-quality RNA elution (60 μL). Five μL of extracted RNA was used to detect SARS-CoV-2 using a SuperScript^TM^ III Platinum^TM^ One-Step qRT-PCR kit (Invitrogen) with the primers and probes described in the CDC protocol (19).

### Statistical analysis

Clinical and demographic information was collected for descriptive statistics. The numerical variables were expressed as mean values ± standard deviation.

## RESULTS

The sample included 50 patients with COVID-19 aged 58±9.9 years on the average. Twenty-four (48%) were female and 26 (52%) were male (Table 1). At the time of ocular sampling, all patients were considered to have a severe form of COVID-19. The average time from onset of symptoms to ocular sampling was 7.6 days (range: 1-15). The main systemic symptoms were cough (62%), shortness of breath (62%), fever (46%) and headache (26%). One patient with extensive ground glass opacity on chest tomography (>50% of lung volume) was in the ICU due to hepatic failure but was asymptomatic at the time of sampling. Forty-six (92%) patients had systemic comorbidities, the most common of which was hypertension (54%), followed by diabetes mellitus (44%), previous or current smoking (30%), cardiovascular disease (24%), obesity (22%) and cancer (18%). All patients had chest computed tomography. The main findings were bilateral ground-glass opacities (96%) and consolidations (58%). None of the patients had ocular signs or symptoms before or during hospitalization.

The rRT-PCR test of the naso/oropharyngeal swabs (taken within 24 hours of the conjunctival swabs) was positive for SARS-CoV-2 in 34 (68%) patients, negative in 14 (28%) and inconclusive in 2 (4%). Patients with negative or inconclusive rRT-PCR results were diagnosed based on other naso/oropharyngeal swabs (n=3) or positive IgG serology (n=13). One conjunctival sample was collected from each patient, totaling 50 samples (25 from the right eye and 25 from the left eye). The conjunctival swabs were positive on rRT-PCR in 5 (10%) patients and inconclusive (amplification of the N1 primer/probe set only) in 2 (4%). The naso/oropharyngeal swabs of the five cases with positive conjunctival swabs (collected within 24 hours) were negative in two cases and inconclusive in one. In these patients, the diagnosis of COVID-19 was later confirmed by IgG serology. The mean CT value of the positive samples was 32.88±2.63 for the N1 primer/probe set and 34.52±3.19 for the N2 primer/probe set (Table 2). The rRT-PCR result of the conjunctival swabs was negative in 43 (86%) samples. Table 3 is a summary of relevant findings from the literature, including the present study.

## DISCUSSION

Five (10%) of the 50 samples of conjunctival swabs from patients with severe or critical COVID-19 were positive on rRT-PCR, and 2 (4%) were inconclusive. Our results confirm the occasional presence of the virus in tear and conjunctival samples. To our knowledge, this is the first Brazilian study to detect the presence of SARS-CoV-2 in conjunctival swabs.

Viral shedding in tears was at first assumed to be secondary to ocular inflammation. Patients with conjunctivitis were therefore expected to display higher rates of positivity for COVID-19. However, several studies have shown that SARS-CoV-2 positivity in the conjunctiva is not necessarily associated with ocular symptoms or signs, as originally believed (6,7,17,20). The efficiency of rRT-PCR analysis depends on the amount of viral RNA in the collected sample (6,7,12,17,21). Early rRT-PCR-based studies found 0-7% SARS-CoV-2 positivity in ocular samples (Table 3). Viral dynamics differ in mild and severe cases of COVID-19, as described by Liu Y et al. (22). Patients with severe COVID-19 tend to have a higher viral load and a longer virus shedding period in nasopharyngeal swabs (22). A systematic review and meta-analysis including 13 studies evaluating serial upper respiratory tract samples showed peak SARS-CoV-2 viral loads inferred from cycle threshold (CT) values within the first week of symptom onset (23). In a comparison of different techniques for tear sample collection using conjunctival swabs and Schirmer paper strips in 75 patients with moderate to severe forms of COVID-19, Arora et al. (17) concluded that conjunctival swabs are best suited for the task. In the study, tear samples were obtained from both eyes and a viral transport medium was used, thus justifying the high rate of positivity reported (24%) (17). In their evaluation of 18 critical COVID patients, Dutescu et al. (7) also found high rates of positivity (28%) for SARS-CoV-2 in tears. The authors concluded that sampling tear fluid after gentle eye massage and one drop of saline solution, using laboratory capillary, may have contributed to the high rates (7).

All 50 patients in our sample had severe/critical COVID infection. Five (10%) conjunctival swabs tested positive, three of which from patients admitted to the ICU, and one death occurred during hospitalization. The conjunctival swabs were collected when the viral load was allegedly high (7.6 days; range: 1-15). This finding is reasonably close to the percentages (7-28.2%) reported in previous studies based on cohorts with more than 10 severe patients (5,6,17,24). To our knowledge, our sample of 50 severe patients is the largest documented so far.

As observed in our study, SARS-CoV-2 may be detected in eyes with no signs or symptoms of inflammation (6,7,13,17,20,25). In a study by Kaya et al. (6), five (16%) out of 32 conjunctival swabs were positive. Two of these had negative naso/oropharyngeal swabs. Both were in the late stage of the disease (18^th^ and 35^th^ day) (6). The two patients in our sample with positive conjunctival swabs and negative naso/oropharyngeal swabs were in the early stage of the disease (9^th^ and 11^th^ day). Further studies are necessary to understand the dynamics of viral load in conjunctival swabs and their relevance to diagnosis and care of COVID patients.

Current diagnosis and surveillance of SARS-CoV-2 depend on rRT-PCR, the result of which is generally given as positive or negative. But the test also provides a semi-quantitative indicator of viral load expressed in CT values which, according to Tom and Mina (26), may be helpful in interpretation and clinical decisions. CT values are not directly comparable between assays, but in general low CT values (high viral load) suggest acute disease and high infectivity, and serial CT values can provide information on the trajectory or subsequent course of illness (26,27,28). A CT value close to the assay detection limit may not be correlated with infectivity since small amounts of RNA could come from nonviable virus (26). While such correlations have been reported for naso/oropharyngeal samples, further studies on larger cohorts are necessary before they can be extrapolated to ocular samples. Two reports evaluating sequential ocular swabs in patients with COVID and conjunctivitis described declining CT values (12,29). Using E gene, RdRp gene and ORF gene as primers, Arora et al. (17) considered samples positive when values crossed the threshold within 35 cycles. In our study we used the N gene-based primers recommended by the CDC, and cutoff was set at 40 cycles (19).

The present study was limited by the small number of samples, the one-time sampling design and the absence of patients with asymptomatic, mild and moderate forms of COVID-19. Moreover, since all patients were evaluated at the bedside, a complete ophthalmological examination was not possible.

In conclusion, SARS-CoV-2 can be detected in ocular samples, raising the question whether the eye is a possible site of primary or secondary infection and a source of transmission in patients with severe disease. The study shows that conjunctival swabs may be positive on rRT-PCR even in the presence of negative naso-oropharyngeal swabs. More research on the interaction between SARS-CoV-2 and the ocular surface and on the diagnosis of COVID-19 based on ocular samples is needed to clarify this point.

## AUTHOR CONTRIBUTIONS

Misawa MAM, Braga PGO and Minelli T helped to design the study, collected samples and drafted and reviewed the manuscript. Yamamoto JH, Tanaka T and Kato JM designed the study and drafted and reviewed the manuscript. Gouvêa MSG conducted microbiology tests and reviewed the manuscript. Pinho JRR reviewed the manuscript.

## Figures and Tables

**Table 1:** Demographic and clinical variables and SARS-CoV-2 RNA detection by rRT-PCR in naso/oropharyngeal and ocular swabs from 50 patients with severe or critical COVID-19.

Patient	Age range (years)	Sex	Result of PCR for SARS-CoV-2 rRT in naso/oropharyngeal and conjunctival swabs	Initial systemic symptoms	Systemic comorbidities	Duration of disease (days)^a^
**Positive conjunctival swab**							
1	60s	M	Negative^b^	Sore throat, cough, asthenia, SOB	CVD, previous alcoholism, smoking	9
2	40s	M	Negative^b^	Cough, fever, SOB	No previous comorbidities	11
3	60s	F	Inconclusive	Cough, nasal discharge, asthenia	CVD, DM, HTN, RA	8
4	50s	M	Positive	Cough, fever, myalgia, SOB	DM	7
5	60s	F	Positive	Chest pain, SOB	Cancer, COPD, DM, HTN, pulmonary thromboembolism	1
**Inconclusive conjunctival swab**							
6	60s	F	Positive	Fever, SOB	Asthma, HTN, obesity	10
7	70s	M	Positive	Cough, fever, SOB	Cancer, CKD, COPD, CVD, DM, smoking, OSA	5
**Negative conjunctival swab**							
8	50s	F	Negative^b^	Cough, fever, SOB	Obesity, previous smoking, schizophrenia	3
9	60s	F	Negative^b^	SOB	COPD, CVD, dyslipidemia, HTN, kidney transplant, previous smoking, overweight	8
10	50s	M	Negative^b^	Cough	DM, HTN	8
11	60s	M	Negative^b^	Anosmia, ageusia, fever, cough, myalgia	No previous comorbidities	10
12	60s	M	Negative^b^	Cough, SOB	DM, dyslipidemia, HTN, previous	9
13	40s	M	Negative^b^	Cough, inappetence, fever, abdominal pain, nausea and vomiting, SOB	CKD, DM, HTN	14
14	50s	F	Negative^b^	Asthenia, headache, inappetence, SOB	DM, fibromyalgia, HTN, obesity	12
15	60s	M	Negative^b^	Anosmia, ageusia, cough	Cancer, HTN, previous smoking	6
16	70s	M	Negative^b^	Cough, nasal discharge, wheezing, headache, myalgia	CVD, DM, HTN, obesity	9
17	50s	F	Negative^b^	Ageusia, hyposmia, headache, cough	No previous comorbidities	9
18	40s	M	Negative^b^	Anosmia, dysgeusia, fever, chest pain, SOB	Obesity	11
19	50s	F	Negative^b^	Anosmia, SOB	DM, HTN	5
20	60s	M	Inconclusive^b^	Headache, fever, myalgia, SOB	Benign prostatic hyperplasia, HTN, obesity	4
21	30s	F	Positive	Cough	CVD, depression, dyslipidemia, DM, HTN, obesity	4
22	20s	F	Positive	Sore throat, headache, fever	Sickle cell anemia	2
23	60s	M	Positive	Asymptomatic	DM, hepatic cirrhosis, HTN, smoking	1
24	80s	F	Positive	Inappetence, wheezing	Actinic pulmonary fibrosis, cancer, Parkinson disease	4
25	30s	F	Positive	Cough, fever, SOB	CKD, HTN, lupus	7
26	40s	M	Positive	Headache, SOB	DM, myasthenia gravis	6
27	60s	F	Positive	Dysgeusia, fever, cough, myalgia	Dyslipidemia, previous smoking	7
28	30s	M	Positive	Cough, fever, diarrhea, SOB	DM	9
29	70s	F	Positive	Inappetence, cough, diarrhea, SOB	CVD, DM, HTN	12
30	50s	M	Positive	Asthenia, cough, chest pain, fever	DM, HTN, kidney transplant	13
31	70s	F	Positive	Hyposmia, headache, cough, nausea, vomiting, inappetence, diarrhea, myalgia, SOB	DM, dyslipidemia, HTN, obesity, previous smoking	8
32	50s	F	Positive	Cough, SOB	Bariatric surgery, cancer	4
33	50s	F	Positive	Fever, diarrhea	Cancer	6
34	70s	M	Positive	SOB	CKD, CVD, DM, HTN, previous smoking	9
35	20s	F	Positive	Cough	A1AD, CKD, granulomatosis with polyangiitis	1
36	70s	F	Positive	Cough, myalgia, SOB	No previous comorbidities	8
37	40s	M	Positive	Anosmia, ageusia, headache, cough, fever, SOB	Cancer	4
38	70s	F	Positive	Cough, nasal discharge, sneezing, headache	CKD, CVD, HTN, previous smoking	8
39	30s	M	Positive	Anosmia, headache, cough, fever, inappetence	Obesity	6
40	60s	M	Positive	Asthenia, nausea, vomiting, SOB	Cancer	3
41	40s	F	Positive	Cough, sore throat, backache, SOB	DM, HTN, obesity	8
42	60s	M	Positive	Ageusia, headache, fever, myalgia	DM, dyslipidemia	12
43	80s	F	Positive	Anosmia, dysgeusia, asthenia, SOB	DM, HTN, sarcoidosis	15
44	60s	M	Positive	Fever, diarrhea, vomiting, SOB	Cancer, HTN, kidney transplant	2
45	50s	M	Positive	Cough, myalgia, SOB	Alcoholism, HTN, previous smoking	12
46	60s	F	Positive	Cough, asthenia, fever, SOB	Cancer, HTN	9
47	80s	F	Positive	Dysgeusia, asthenia, headache, fever, SOB	Asthma, COPD, DM, HTN, obesity, depression, OSA, previous smoking	10
48	30s	M	Positive	Cough, headache, fever, myalgia, SOB	No previous comorbidities	9
49	70s	M	Positive	Cough, fever	CVD, HTN, previous smoking and alcoholism	8
50	60s	M	Positive	Cough, myalgia, SOB	Liver transplant	15

A1AD=alpha 1 antitrypsin deficiency; COPD=chronic obstructive pulmonary disease; CKD=chronic kidney disease; CVD=cardiovascular disease; DM=diabetes mellitus; F=female; HTN=hypertension; M=male; RA=rheumatoid arthritis; SOB=shortness of breath; OSA=obstructive sleep apnea; ^a^Duration from onset of symptoms to collection of ocular sample; ^b^all naso/oropharyngeal swabs negative or inconclusive on RT-PCR had COVID-19 diagnosis confirmed by another RT-PCR test or serology.

**Table 2:** Comparison of rRT-PCR results for SARS-CoV-2 in naso/oropharyngeal swabs and conjunctival swabs from COVID-19 patients with viral detection in ocular samples.

			Conjunctival swab		
Patient	Duration (days)	Naso/Oropharyngeal swab	CT N1	CT N2	
1	9	Negative	30.8	33.7	Positive
2	11	Negative	32	31.4	Positive
3	8	Inconclusive	31.6	31.6	Positive
4	7	Positive	37	35.9	Positive
5	1	Positive	36.2	40	Positive
6	10	Positive	36.9	Undetermined	Inconclusive
7	5	Positive	36	Undetermined	Inconclusive

CT=cycle threshold (in cycle numbers).

**Table 3:** Summary of studies using rRT-PCR to detect SARS-CoV-2 in ocular samples from patients with COVID-19.

Author	Ocular symptoms (n)	Patients (n)	Positive rate (%)	Form of disease
Xia et al. (13)	1	30	3	Common type (n=21)
				Severe (n=9)
Liang et al. (4)	3	37	2.7	Mild (n=25)
				Severe (n=12)
Zhang et al. (12)	2	72	1.4	Not reported
Seah et al. (9)	1	17	0	Not reported
Wu et al. (2)	12	38	5.3	Moderate (n=4)
				Severe (n=2)
				Critical (n=6)
Xie et al. (30)	0	33	6.1	Not reported
Fang et al. (31)	Not reported	32	15.6	In ICU (n=8)
Karimi et al. (24)	1	43	7	Severe (n=43)
Atum et al. (21)	10	40	7.5	Not reported
Kumar et al. (25)	0	45	2.2	Not reported
Guemes-Villahoz et al. (15)	18	36	5.5	Mild (n=17)
				Moderate (n=12)
				Severe (n=7)
Arora et al. (16)	0	75	24	Moderate (n=36)
				Severe (n=39)
Shemer et al. (10)	3	16	0	Not reported
Kaya et al. (6)	0	32	15.6	Mild (n=5)
				Severe (n=22)
				Critical (n=5)
Mahmoud et al. (8)	10	28	28.6	Mild/moderate (n=25)
				On ventilator (n=3)
Dutescu et al. (7)	0	18	27.7	In ICU (n=5)
Zhou et al.(14)	1	67	4	Not reported
Savastano et al. (17)	5	50	8	Hospitalized and ICU patients
Yan et al. (20)	0	35	8.6	Mild/moderate (n=29)
				Severe (n=6)
Present study	0	50	10	Severe (n=50)

ICU=intensive care unit.
